# Development and Management of a Geographic Information System for Health Research in a Developing-country Setting: A Case Study from Bangladesh

**Published:** 2007-12

**Authors:** Jonathan D. Sugimoto, Alain B. Labrique, Ahmad Salahuddin, Mahbubur Rashid, Rolf D.W. Klemm, Parul Christian, Keith P. West

**Affiliations:** 1 Center for Human Nutrition, Department of International Health, Johns Hopkins Bloomberg School of Public Health, Room W2505, 615 North Wolfe Street, Baltimore, MD 21205, USA; 2 JiVitA, House 63, Road 3, Karanipara, Rangpur, Bangladesh

**Keywords:** Geographic information systems, International health, Nutrition, Medical informatics, Community health research, Community mapping, Bangladesh

## Abstract

In the last decade, geographic information systems (GIS) have become accessible to researchers in developing countries, yet guidance remains sparse for developing a GIS. Drawing on experience in developing a GIS for a large community trial in rural Bangladesh, six stages for constructing, maintaining, and using a GIS for health research purposes were outlined. The system contains 0.25 million landmarks, including 150,000 houses, in an area of 435 sq km with over 650,000 people. Assuming access to reasonably accurate paper boundary maps of the intended working area and the absence of pre-existing digital local-area maps, the six stages are: to (a) digitize and update existing paper maps, (b) join the digitized maps into a large-area map, (c) reference this large-area map to a geographic coordinate system, (d) insert location landmarks of interest, (e) maintain the GIS, and (f) link it to other research databases. These basic steps can produce a household-level, updated, scaleable GIS that can both enhance field efficiency and support epidemiologic analyses of demographic patterns, diseases, and health outcomes.

## INTRODUCTION

A geographic information system (GIS) is a digital cartographic database containing the locations and characteristics of features within a geographic region, with additional data about events and conditions occurring at these locations. Over the past two decades, community health research has begun to adopt GIS for epidemiologic investigations. Primarily, these investigations have mapped and modelled the spatial occurrence of infectious (1–3) or non-infectious diseases (4–6) and their covariation with environmental risk factors (7–9). Less often, GIS has been used for modelling healthcare-seeking behaviours and preventable adverse health outcomes with distances to healthcare facilities and providers (10–12).

Most health research incorporating GIS has been conducted in higher-income countries (13,14) where digital, routinely-updated, geographically-referenced health data typically exist (15). Increasingly, studies in lower-income countries are incorporating GIS into their design (1–3,5–8,12), but rarely report more than a brief overview of the methods and equipment used during development (1,16–19). For example, Emch and colleagues describe the development of a medical GIS designed to interface with the Matlab demographic health surveillance system of ICDDR,B (20,21). A combination of satellite imagery, aerial photography, and field validation surveys was used for producing this GIS. Ali *et al.* reported the development of a GIS encompassing four peri-urban slums of Karachi, Pakistan (22), designed from satellite imagery and field validation surveys to study the epidemiology of vaccine-preventable diseases in urban South Asia. Hightower *et al.* described the construction of a GIS in rural Kenya—containing the locations of houses, roads, landmarks of interest, and the potential breeding sites of the malaria mosquito vector—using differential GPS survey techniques (23). Using radio communications equipment, professional-grade global positioning system (GPS) receivers, a personal computer, and vehicles, three surveyors developed a GIS for their 70-sq km study area in four months. These health studies which have incorporated GIS remain the exception, rather than the norm, as perceptions about high costs, technical complexity, and difficulty of use continue to prevent the widespread incorporation of GIS methods as standard field research tools.

**Fig. 1:**
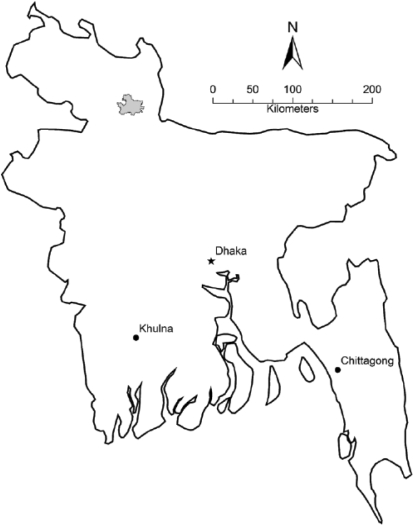
JiVitA study area

There remains, therefore, a need for community health researchers to appreciate the steps involved in building and maintaining a basic research GIS, given recent hardware and software advances that have significantly increased user-friendliness and reduced costs. This report describes a framework for developing and maintaining a scaleable GIS, drawing on practical experiences in developing and applying a system in northern Bangladesh. The system was developed to serve a research trial site covering a 435-sq km rural area comprising one-fifth of the land mass of northwestern Gaibandha district and ∼1% of adjacent Rangpur district, with an estimated population of 650,000 and a population density of ∼1,500 per sq km (Fig. 1). The shape and size of the site emerged from the need to have a contiguous rural area, excluding urban and rural growth centres, with a population sufficient for a series of community randomized trials evaluating the effects of maternal and newborn nutritional supplementation on outcomes such as mortality and morbidity (The ‘JiVitA Project’ (24)). The first trial (JiVitA-1, Clinicaltrials.gov# NCT00198822), which began in mid-2001, was designed to evaluate the impact of weekly maternal vitamin A or beta-carotene supplementation on maternal and infant mortality, requiring a sample size of over 68,000 incident pregnancies followed through six months postpartum. A second, nested, trial (JiVitA-2, Clinicaltrials.gov# NCT00128557) aimed to assess the effect of vitamin A supplementation immediately after birth on reducing infant mortality by enrolling 25,000 newborn infants. These trials have maintained a pregnancy surveillance system which requires that each of ∼150,000 houses be visited every five weeks by a team of 650 field workers.

The JiVitA study area (grey shading) located in northern Bangladesh (outline), with capital (star) and major cities

The large size of the project area, high density of population, and frequent contact with participants over an extended period of time required an efficient participant-tracking system, with a geographic component that would spatially relate and display addresses, other relevant points, e.g. field offices or healthcare facilities, and events of interest, e.g. pregnancies, births, and deaths. These requirements were readily met by converting and updating conventional paper maps with GIS technology. The steps between paper maps and a digital GIS are outlined below and can serve as a framework for other health researchers. The order and necessity of these steps, however, depend largely on the materials publicly available at the beginning of a new study.

## MATERIALS AND METHODS

A fully-operational GIS in northern rural Bangladesh was produced in six stages, namely, by (a) updating and digitizing existing paper maps; (b) joining the digitized maps into a single contiguous map of the project area, called a ‘basemap’; (c) referencing this basemap to an accepted geographic coordinate system; (d) collecting and inserting the geographic locations of participant residences and other landmarks of potential interest; (e) continuously updating the GIS to reflect the dynamic nature of a free-living population; and (f) linking the GIS to research data to enhance project operations and optimize information capture. The six stages are described below.

**Stage 1: Updating and digitizing existing paper maps**

***Obtaining paper maps:*** At the beginning of the JiVitA project, neither paper nor digital maps depicting the entire study area in sufficient detail were publicly available. The study area-mapping process, thus, began in 2000 with the purchase of 297 public-domain smaller, local-area land allocation paper maps drafted from aerial photographs taken during the early 1930s. These cadastral (land registry) maps were the result of extensive land-ownership surveys by the British Survey of India. Each 1:3960-scale map covers a land area of 0.5 to 4 sq km, including land plot numbers and boundaries, local roads and paths, streams, fields, and communities (e.g. neighbourhoods and villages with administrative boundaries as they existed at that time). Surprisingly, the land and political boundaries depicted on these 70-year old maps were sufficiently accurate to stitch together a contiguous study area map, serving as a foundation for the GIS.

***Field validation survey:*** The paper maps were updated via an extensive field-validation survey conducted as part of a household enumeration census. Sixty-four trained two-member teams visited each house within the boundaries of a single British-era cadastral map sheet, enlarged by photocopying to facilitate field work. Each team assigned unique house numbers (addresses, required for study subject identification and follow-up), painted the numbers on robust household surfaces and recorded (not to scale) approximate house locations on the enlarged photocopies. New and updated locations of roads, rivers, and other landmarks, e.g. weekly market places, mosques, and clinics, were drawn on the paper maps using standardized map symbols. Supervisors resurveyed ∼1% of the houses to verify assigned household locations and address-assignments. These maps, now containing approximate household locations as placeholders, allowed a smaller GPS-equipped team to conduct the GPS survey described below in stage 4.

**Fig. 2:**
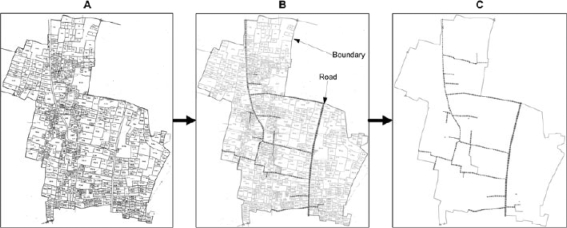
Stage 1—Updating and digitizing existing paper maps

***Converting the paper maps to a digital format:*** At the same time as the field teams were updating the photocopied, enlarged maps, the original 297 cadastral maps were computer-scanned to a digital picture file format (TIFF Group 4) and converted to a line (vector) format using the digitizing tools of AutoCAD Map 2000 (Autodesk Inc., San Rafael, CA) and the automated tools of the AutoCAD extension, CAD Overlay 2000 (Autodesk Inc., San Rafael CA) (25) (Fig. 2). When the scanned copy of each map was imported into AutoCAD Map, the geographic scale was first defined using a scale bar in the map's legend. Administrative boundaries, e.g. villages, were automatically traced (vectorized) using CAD Overlay. Roads, rivers, and canals were manually traced using the standard AutoCAD Map drafting tools. We differentiated landmark types by storing each type in a separate colour-coded layer within a map's AutoCAD file. Once the hand-updated, photocopied paper maps were returned by the field survey teams, GIS technicians inserted houses and updated drawings of roads and rivers.

(A) Scanned original copy of a 1930's cadastral local-area map. (B) Key linear features (i.e. roads and boundaries) were traced, essentially ‘removing’ undesired content. (C) Linear features and landmarks in each map were updated with new data to form a ‘new’ digitized map

**Stage 2: Joining the digitized maps into an area basemap**

**Fig. 3:**
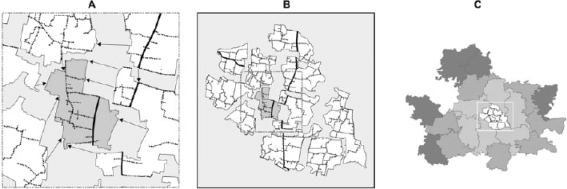
Stage 2—Joining the digitized maps into a basemap

One utility of a GIS is the ability to display and analyze data on an extremely local-area scale (e.g. a cluster of morbidity at a village level or a specific field team's work area), but also on a wider large-area scale (e.g. a whole study area, comprising multiple clusters or worker catchment areas) which provides a wider context for localized or clustered events. This is only possible if the digital maps for each local area have been joined into a single map, known as a ‘basemap’. For JiVitA, the 297 AutoCAD digitized local-area maps were pieced together using a two-phase, jigsaw-puzzle approach (Fig. 3). The local-area maps were first joined to form 19 union-level maps that were subsequently joined into a complete basemap of the study area.

(A) Adjacent digitized local-area maps are molded to the central (anchored) map (darkly-shaded area, from Fig. 1) using rubbersheeting points (arrows) from each local-area map. (B) The digitized local maps are rubbersheeted into a single map of an administrative union. Paved road (); Dirt road (). (C) Repeating this process for each union, the resulting union maps are rubbersheeted together into a single basemap of the JiVitA project area, beginning from the centre and working outward (indicated by darker levels of shading)

***Rubbersheeting and cleaning:*** Each time adjacent maps were joined along their shared boundary using a process known as rubbersheeting and cleaning. Rubbersheeting is a non-linear adjustment algorithm (25) implemented by AutoCAD Map. One of the two maps being joined was designated the ‘anchored map’, unaltered, to which the second map's boundary was molded. Points along the boundary of the unanchored map were matched with corresponding locations along the boundary of the anchored map, forming pairs of points. The rubbersheeting algorithm molds the unanchored map to the anchored map by snapping the selected points along the boundary of the unanchored map to the corresponding point in the anchored map. The locations of the remaining features of the unanchored map were adjusted to maintain their position relative to each other. This can lead to slight geographic distortions in the second, unanchored map. We attempted to minimize these distortions by starting with a central local-area map of a larger area, e.g. Union, and rubbersheeting adjacent maps in circular layers, radiating out towards the edges of the larger area. During the rubbersheeting process, duplicate copies of boundary lines, road segments, point landmarks, river segments, and other map features (one copy from each of the two separate maps) were occasionally found along a shared boundary. We removed any duplicates, leaving a single feature on the central anchored map.

***Editing the new basemap:*** To maximize the utility of road data in a GIS to guide field operations or for epidemiologic analyses, the lines representing roads should form a continuous network across local-area maps. Specifically, no gaps should exist between intersecting line segments. Many different scenarios had to be considered, and standard rules had to be decided upon for resolving conflicts consistently, such as joining roads and paths that spanned adjacent maps. For JiVitA, once all local-area maps had been joined into a single basemap, the line-gap detection functions of AutoCAD Map 2000 (33) were applied to intersecting road data to detect and fix any gaps.

After rubbersheeting and cleaning, the basemap was converted into a format compatible with ArcGIS ArcView (version 8.1, Environmental Systems Research Institute, Redlands, CA), a software that offers an intuitive editing interface for the background attribute data that describes each mapped landmark (e.g. feature type, length, and size).

**Stage 3: Referencing the basemap to an accepted geographic coordinate system**

An advantage of using a GIS over traditional, image-only maps is its capacity to efficiently integrate data from diverse sources—external and internal to a project—into a single system. Inserting data collected by GPS or other external sources into a basemap requires that all data points be stored in geographic coordinate systems that reference the same map datum (13). A geographic coordinate system is a two-dimensional grid that models the three-dimensional curvature of the surface of the earth. Each coordinate system is defined by its X (longitudinal) and Y (latitudinal) axes and a nearby ‘map datum’, which is a prominent geographic feature on the earth's surface relative to which all other X,Y-coordinates of the system are calculated (13). The nearest prominent geographic feature to our project area is Mt. Everest, so JiVitA GIS data are stored in a geographic (latitude/longitude) coordinate system (GCS) using the Everest 1830 map datum (26). For analyses requiring metres as a distance unit, the Bangladesh Transverse Mercator (BTM) projection (26) was employed. Since BTM references the same map datum as the chosen GCS, ArcGIS can seamlessly translate GIS data between the two systems.

***Georeferencing the basemap using ground control points:*** Although most commercially-available GIS-ready basemaps are referenced against global geographic coordinates, a newly-created GIS basemap may need to be contextually situated on the Earth's surface, or georeferenced. Here, independently-measured geographic coordinates (e.g. obtained by GPS or external map sources) of prominent features in the study area—known as ground control points (GCPs)—are matched with their corresponding location on the project GIS maps. A non-linear adjustment algorithm (similar to the AutoCAD rubbersheeting algorithm) is applied to fit the rest of the basemap around this framework of GCPs (13). Adjusting the rubbersheeted basemap over a scaffolding of georeferenced GCPs also served to correct any warping caused by the stitching process.

**Fig. 4:**
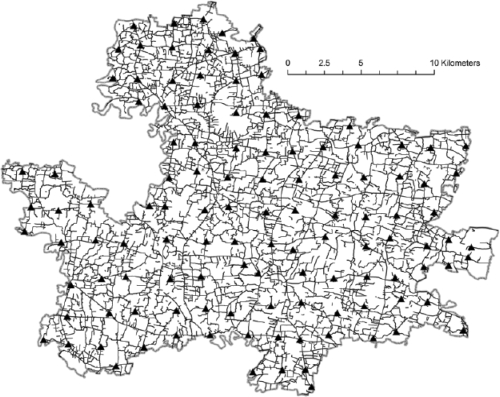
Stage 3—Referencing the basemap to an external (global) coordinate system

Major road intersections were chosen as the GCPs to georeference the JiVitA basemap (Fig. 4). GPS receivers (Etrex Venture, Garmin International Inc., Olathe, KS) were used to collect the coordinates for these GCPs. A 2-by-2 km grid (chosen to balance accuracy and resource considerations) was overlaid on the basemap, and the nearest road intersection to the each grid point was selected as a GCP. Road size, permanence, and the accessibility of the intersection were also factored into our selection process. One of the authors (JS or SA) and an experienced member of the field staff of the Project surveyed each of these road intersections. Error of GPS measurement was minimized using the geometric mean of three measurements (mean measurement-to-GCP distance of 2.2 metres, standard deviation [SD]=3.0) taken at each location to represent the final coordinates for each corresponding GCP. Using the Spatial Adjustment extension in ArcGIS (version 8.3, Environmental Systems Research Institute, Redlands, CA), the GCP locations in the basemap were matched with and then snapped to their corresponding GPS-measured coordinate.

Coordinates for a network of immobile geographic features, such as road intersections (▴), are collected using global positioning system (GPS) receivers. To improve precision and accuracy, the geometric mean of three GPS measurements taken at the same location defines the final coordinate for each road intersection. Roads (); Study area boundary ()

***Fine-tuning the georeferencing:*** A scarcity of road-intersections (GCPs) near the boundaries of the project area can lead to distortions being introduced during the rubbersheeting process (Stage 2) that are not adequately adjusted near the perimeter of a project area. To minimize the effect of these distortions and enhance overall GIS accuracy, the locations of bridges, over which roads pass, were collected during a GPS survey of point landmarks (described below) and used in a second round of georeferencing. To do this, the nearest point along a road was moved to coincide with the GPS location of each bridge. Bridges located too far from any road segment (>150 m) were excluded from this process as data anomalies. Throughout this iterative process, all locations previously used as georeferencing points, including the GCPs, were defined as anchor points. To retain the integrity of preceding georeferencing adjustments, map features at these anchor points were not permitted to move.

**Stage 4: Adding residence and landmark locations to the GIS**

**Fig. 5:**
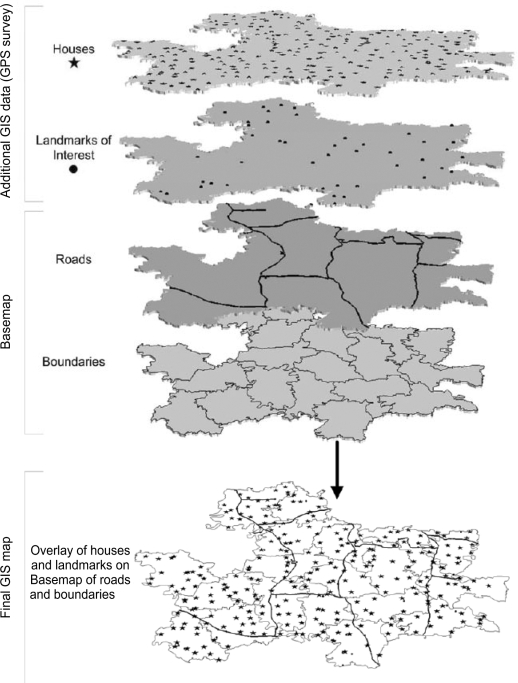
Stage 4—Adding locations of houses and other landmarks to the GIS

To facilitate tracking of current and future community trial participants and accurately locate houses, all residences within the JiVitA Project area were surveyed using handheld GPS receivers (Etrex Venture, Garmin International Inc., Olathe KS). Other landmarks of environmental, social, public health and economic interest across the Project area were also surveyed by GPS at this time, added to the GIS, and overlaid onto the basemap to form the final JiVitA Project GIS (Fig. 5).

Locations (coordinates) of houses (⋆) and landmarks of interest (•)—surveyed by GPS—are overlaid on the basemap layers to form the final GIS map. For display purposes, the number of locations shown has been limited

***The GPS survey:*** During the construction of the JiVitA GIS, a small team of six surveyors and one supervisor employed preprinted household number lists, landmark location-recording forms, and handheld GPS receivers [A GPS receiver uses radio signals transmitted from four of 21 orbiting satellites to calculate, via trilateration, its location and elevation on the surface of the earth (27)] to visit and record the geographic coordinate of every house (∼138,000) and point landmark of interest within the study area (∼87,000). Point landmarks collected included health service providers, shops, mills, non-governmental organization offices, ponds, bridges, tubewells, and others (Table). For quality-control purposes, 2.0% of the total houses surveyed (n=2,710) were independently resurveyed by the team supervisor. Under 3% (n=77) of the resurveyed houses by the supervisor were different from the surveyor's measurements by >40 metres (>2 times our allowed positional inaccuracy limit of 20 metres, itself chosen due to the inherent 10-metre maximum resolution of our handheld units). Those so identified during the quality-control round were resurveyed.

**Table:** Geographic profile of the JiVitA Project in northern Bangladesh: point, linear, and area features∗

Point features	
Landmark type	No.

Households	149,402
Tubewells	56,406
Lakes or ponds	23,008
Shops or markets	3,456
Religious facilities	1,829
Schools	1,087
Agricultural production facilities	795
Healthcare service providers	603
Non-governmental organizations	95

Total no.	236,681

Line features	
Landmark type	Cumulative length (km)

Railroads	27.7
Paved roads	126.6
Unpaved (dirt) roads	1,667.0
Footpaths	234.9

Total kilometres	2,056.2

Area features	
Area type	Cumulative area (sq km)

Project area	435


∗Geographic profile based upon the status of the JiVitA GIS at the end of June 2006

**Stage 5: Maintaining the GIS**

Given the dynamic nature of rural community life, it may be desirable (if the uses of the GIS, personnel, and funds so allow) to maintain a developed GIS by regularly updating the addition, movement, and destruction of landmarks of interest, especially the houses of study participants. Given the common use of impermanent house-construction materials (bamboo, thatch, mud, wood, and tin) in this area of Bangladesh, we expected and found a high rate of house turnover (new construction, house relocations, destroyed houses). On average, we see ∼10,000 new houses built each year in this area of Bangladesh. Due to this high volume of change and the logistical limitations of working with such a large study area, the JiVitA GIS is continuously being updated, accurately reflecting the count and location of all landmarks, at most, two months behind reality. It is important when conducting spatial analyses to ensure that the location of an outcome and the spatial factors being related to that outcome (i.e. distance to facilities, source of infection, proximity to a main road) reflect the spatial location(s) at the time the outcome actually occurred. (e.g. An analysis of immunization campaign success rates by proximity to vaccine-delivery centres must have GIS data on household locations of children at the time the campaign was conducted, for best results.)

The Project has developed a two-phase GIS updating system that, in part, nests collection of house or landmark updates into the routine weekly data collection for existing field activities. First, new, moved, or destroyed houses or landmarks are reported on a weekly basis by the field staff of the Project through a vital events reporting system that is part of our ongoing trials. These field reports are entered into the Project database, and within a week, a list of new and moved houses scheduled to be surveyed is sent to the five-member, field-based GPS survey team. Using this list, a team member routinely visits each part of the study area every two months, using GPS receivers to record the current geographic location for the new and moved houses on the list. The resulting GPS data and completed lists are returned to the Project data-management centre and merged into the database, thereby updating the location of each house in the GIS.

**Stage 6: Linking and using the GIS database**

**Fig. 6:**
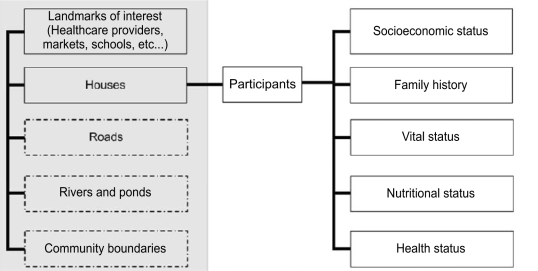
Stage 6—Linking and use the GIS database

The research utility of a GIS lies with its ability to link to other project databases (Fig. 6), providing a spatial dimension to otherwise ‘flat’ (nongeographic) participant health, socioeconomic and outcome data. We record the address (study-defined neighbourhood code, plus household number) of each participant at enrollment, or upon movement to another household, providing the database link between health-outcome data for each participant and the location of their household in the GIS.

The ‘community boundaries’ GIS component includes multiple levels of government and JiVitA-defined administrative regions. Links (——) between GIS (shaded grey) and project database components are shown, with each participant serving as the central link between the project database and the GIS. Data are stored in a proprietary ArcGIS (ESRI, Redlands, CA) database format, a geodatabase (dotdash box outline), and a relational database management system, SQL server 2000 (Microsoft Corp., Seattle, WA) (solid box outline)

This linkage also allows the GIS to be used for increasing the efficiency of field research operations. For example, we built a simple GIS interface which allows data collectors to create GIS ‘guide’ maps or even upload the coordinates of houses scheduled for interviews into a handheld GPS. In rural settings where houses are often randomly placed in clusters of varying size, finding individual houses is a challenge for people from outside the community. The use of GPS resulted in a one to two-fold increase in time efficiency, which is cost-effective for high-level field workers, such as research physicians. The linkage also enhances the research capacity and scientific yield of a study, allowing the quantification of spatial relationships with the occurrence of disease, pregnancy outcomes, mortality, etc. That is, not only looking for spatial clusters or patterns in the location of certain types of outcome, but extending analytic capabilites to explore the effect of proximity to landmarks of public-health interest on outcomes and behaviours (e.g. the relationship between clinic location and treatment-seeking behaviour/postpartum care/pregnancy-related morbidity and mortality). The ability to also adjust for the potential influence of neighbours (e.g. spatial autocorrelation, or when a value at any point in space is dependent on values of surrounding points) in analyzing health outcomes is also a valuable benefit of a research GIS.

## DISCUSSION

Geographic information systems have the potential to be integral components of community-based health research and service-delivery programmes in low-income countries, providing spatial information to guide field operations and improve epidemiologic investigations. In recent years, although we have seen an increased number of studies using GIS to improve our understanding of the health epidemiology and disease dynamics (1,3,7,17,18,28,29–32,35), few have described in detail how GIS resources can be developed, organized, and maintained (20–23), especially under resource-poor, remote, or rural conditions.

As GPS and other digital cartographic technologies advance rapidly, there remains a basic need to describe and lay out the practical steps required to develop and use the GIS to support health research and delivery of services. In this report, we have described six basic stages of setting up and maintaining a GIS in a circumscribed rural area of northern Bangladesh, a setting for which no pre-existing digital maps were available. These stages represent our experiences in combining low-cost, state-of-the-art digital mapping tools with field research capabilities to create a GIS intended to improve operational efficiency, enhance participant tracking for longitudinal follow-up, and provide a new platform for geospatial investigation in community nutrition and health research. The stages we have described are broadly generalizable, although their direct utility to other settings will depend on many factors, including the size of the study area to be mapped, the level of detail needed (types of landmarks and coordinate precision), and the availability and quality of existing maps. Also important are the project's manpower, mobility, existing data-collection capabilities, and the level of sophistication of available computers, software, data entry, and management systems.

The above-described ‘first stage’—to update and digitize existing maps—presupposes an availability of paper maps that are reasonably accurate with respect to scale and boundary definition. This requirement is often not met (36–38), leading to a need to draw detailed paper maps of a study area at the outset of a project that could later be digitized. Increasingly, remote-sensing products, e.g. satellite and aerial photography, are available to researchers and community programmes that, while usually of lower resolution and incomplete with regard to information required for a project, could be adapted for use as a basemap. When pre-existing scaled maps with acceptable boundaries are available, irrelevant or imprecise content can be ‘removed’ by only tracing desired lines, (e.g. roads and streams), and other features when digitizing these maps.

The size of the project area and the number of smaller local-area maps determine the extent of rubbersheeting required. We were able to identify and purchase 297 local-area maps drawn seven decades ago from aerial photography, each representing a few square kilometres, from the Land Records Office of the Government of Bangladesh (at a cost of ∼$4 each). These maps provided the basic size and boundary details for the JiVitA study area that, once verified and updated, contributed the basic structure of our GIS product.

Georeferencing a project area map to a standard geographic coordinate system permits new GIS data obtained in-house, or from other agencies, e.g. United Nations Children's Fund, local or federal government sources, etc., to be superimposed accurately onto the project maps. Although we used road intersections as ground-control points (GCPs), any immobile feature that is identifiable on study maps and in the real world can be used for this purpose. Assuming these conditions, features that approximate a single geographic point, e.g. intersections of roads, offer the best options as GCPs.

Inserting and routinely updating locations of residences improve operational efficiency in the field by enabling supervisors, physicians, and other highly-skilled staff, who often need to cover large areas on motorcycle or bicycle, to rapidly find target houses for interviews and assessments. Typically, digitized maps can be printed daily or weekly for such staff, indicating homes to visit, outlining routes to travel, and providing locations of offices, clinics, and other features that can enhance field performance. Entering the geographic coordinates of health clinics, markets, and other community landmarks into the GIS maps subsequently enables spatial analysis of associations between health status, outcomes, and behaviours with location and use of healthcare and other facilities in the area. In JiVitA, we have also used the GIS for monitoring compliance and identifying clusters of high noncompliance for targeted community advocacy.

To use the GIS for research and operational analysis, features in this database should be linked to data of participants stored in other project databases. In a dynamically-updated environment, storing the geographic coordinates of point landmarks using the same relational database software can increase the efficiency of linking the GIS and project databases. These links can also be dynamically updated by defining them as simple queries, which are re-run as needed.

In addition, prior to using GIS-derived data for research analysis, geographic precision of the system must be accurately estimated. Although this step was not included with our six-stage process, preliminary estimates of the basemap and accuracy of the GPS survey were derived from GPS measurements of road intersections (n=7) and duplicate measurements of households (n=2,710) respectively. They were 30.3 metres (standard deviation [SD]=22.1) and 14.0 metres (SD=32.8) respectively. A more formal assessment of the accuracy of this system will need to be completed in the future.

The building and maintenance of a GIS in resource-poor settings is now within reach of community-based research and intervention programmes in the developing world. Advances in user-friendly, inexpensive technology allow the incorporation of GIS as a tool to enhance science and implementation efficiency with minimum additional investment beyond the cost of setting up studies or intervention projects.
